# Special Issue: “Novel Diagnosis and Treatment of Gastrointestinal Disease”

**DOI:** 10.3390/life14121603

**Published:** 2024-12-04

**Authors:** Doina Georgescu, Felix Bratosin

**Affiliations:** 1 Department of Internal Medicine I Medical Semiotics I, Center for Advanced Research in Cardiovascular Pathology and Hemostaseology, “V Babes” University of Medicine and Pharmacy, 300041 Timisoara, Romania; 2 Department of Infectious Diseases, “V Babes” University of Medicine and Pharmacy, 300041 Timisoara, Romania; felix.bratosin@umft.ro

Significant advances in the diagnosis and treatment of gastrointestinal diseases have been made in recent years. The breadth of medical knowledge, specifically on gastroenterology, is growing exponentially. However, there is still much to be understood and clarified and there are still many questions to be answered; in the future, the solutions to these questions may revolutionize our approach to medicine, not only in terms of diagnoses but also regarding therapy [1].

The main aim for this Special Issue is to emphasize both the most innovative and reliable approaches for gastroenterological issues, which encompass a wide variety of conditions and diseases.

The prospective study by Aloysius, M. et al. [2] aimed to assess whether the overall polyp detection rate (PDR), as a clinical asset, can be a reliable surrogate for colonoscopy screening, by comparing PDR to the “gold standard” indicator, the adenoma detection rate, in order to evaluate the efficacy of screening. of the study showed strong correlations between the overall PDR and the screening, surveillance, and diagnostic. The authors concluded that PDR may be used in clinical practice as a reliable surrogate for screening.

In their original study, Groza, A.L. et al. [3] addressed the challenges related to prevention strategies for colorectal carcinoma (CRC), specifically regarding diminutive polyps. The study illustrated an original prevention strategy aimed at developing a combination of strategies such as resect-and-retrieve or resect-and-discard, resulting in a decreasing necessity for an optical diagnosis. This tailored diminutive polyps-based approach might optimize not only CRC prevention but also intervention practices.

Miutescu, B. et al. [4] investigated, in their retrospective study, a potential life-threatening and resource-consuming situation, concerning the multidrug-resistant clinical forms of acute cholangitis. The study aimed to investigate whether previous specific interventions, such as sphincterotomy, with or without stent placement, may affect the clinical and microbiological characteristics of acute cholangitis. The results of the study highlighted that patients for whom both procedures had been performed (sphincterotomy and stent placement) were at an increased risk for infections with multidrug resistance patterns.

In their preliminary cross-sectional study, Mateescu, T. et al. [5] explored the burden of anal fistulas in terms of health-related quality of life and associated stress-related disorders, by comparing two groups of patients with or without inflammatory bowel disease (IBD), who completed various questionnaires at their initial admission and three months post-procedure. As hypothesized, the IBD group showed significant improvement in their physical, mental, and total health scores. In terms of mood disorders, however, the non-inflammatory group displayed a more significant decrease in anxiety and depression scores.

Melita, G. et al. [6] carried out a narrative review on conducting moderate or deep sedation during endoscopic retrograde cholangiopancreatography, considering the fact that, to date, no guidelines concerning the level of sedation during this procedure are currently available. Taking into account the different opinions of the scientific community on this topic, the authors decided to search the current literature, in order to highlight the advantages and disadvantages of the various sedation types and to display evidence that supports the effectiveness of one type over another. They found that moderate sedation with Dex seemed to be successful, demonstrating not only good efficiency during the procedure but also enabling the rapid recovery of the patient after the endoscopy.

In their review, Faur, A.C. et al. [7] addressed uncommon neoplasms, such as salivary glands tumors, which are known for having heterogeneous epidemiology and unique pathological features, as well as high clinical unpredictability. The study emphasized the importance of histological imaging-based diagnosis, highlighting new advances in the field, including the assistance of artificial intelligence and new treatments.

Facchin, S. et al. [8] presented an overview of the particularities of the gut microbiota in terms of metabolic performance. The study exhaustively investigated insights from the physiology of short chain fatty acids (SCFAs), such as acetate, propionate, and butyrate, as well as the important roles they play, not only in the promotion of healthy digestive processes, but also in contributing to metabolic homeostasis. This review also emphasized the current therapeutic implications of SCFAs.

In their review, Woodhead, G. et al. [9] focused on advancements in therapy for locoregional intrahepatic cholangiocarcinoma (iCCA), not only in cases of early diagnosis and resectable tumors, but also in advanced stages of diagnosis. The study highlights the importance of locoregional therapies, drawing attention to the increasing role of interventional radiology with liver-targeted therapies.

Ardeshna, D.R. et al. [10] reviewed the position of microwave ablation in the treatment of different pathological forms of pancreatic cancer, after comparing this particular procedure to radio frequency ablation (RFA). Although some concerns related to peri-procedure complications still exist, the study concluded that microwave ablation has exhibited comparable results with other current and efficient techniques in the treatment of pancreatic tumors.

In their review, Belei, O. et al. [11] focused on understanding the role of gut microbiota in celiac disease and on profiling the most reliable therapies for this condition. They found that modulating the gut microbiota in patients with celiac disease may represent an important asset in restoring the local microbiome homeostasis and normal gut permeability, as well as in mitigating the immune response.

In their case report, Ciobica, M.-L. et al. [12] presented an atypical case of asymptomatic cholangiocarcinoma (CCA) that demonstrated an onset with paraneoplastic syndrome (Trousseau Syndrome), characterized by recurrent superficial vein thrombosis in a patient already taking anticoagulants. The presented patient exhibited many particularities, such as multiple episodes of SARS-CoV-2 infection and a history of bariatric surgery. The authors also took the opportunity to present a brief literature review of this challenging topic.

Rosca, C.I. et al. [13] have drawn attention to the etiology of chest pain as one of the most usual causes for ambulatory care as well as for emergency room visits. Their case report highlighted that chest pain may be due to various causes, but they warn to never underscore this symptom, as it is often associated with coronary ischemic disease, even in patients where a different diagnosis has already been made. In this particular case, the diagnosis was a leiomyoma of the esophagus.

Georgescu, D. et al. [14] addressed the diagnosis and treatment of hepatic veno-occlusive disease (VOD), a rare vascular condition of the liver, in an atypical, late-onset case of a female patient treated for colorectal carcinoma using chemotherapy. Considering the difficulties and pitfalls of VOD therapy, the authors reiterated the importance of the early diagnosis of hepatic VOD in patients treated with drugs of risk.

Albu, T.A. et al. [15] presented original imaging features and personal observations related to a case of a primary hepatic angiosarcoma. The authors focused on describing the radiological characteristics of this rare and extremely aggressive liver tumor, proposing a second later magnetic resonance imaging( MRI )T1-weighted scan that may improve the accuracy of the diagnosis.

In their case report, Ivan, V.S. et al. [16] presented a critical situation wherein an elderly male patient, presenting with an acute myocardial infarction which was treated using primary percutaneous intervention, who developed early post-stenting hemorrhagic complications, prompting challenging therapeutic and diagnostic decisions. The patient was found to have not only a severe heart condition but also two associated malignant tumors, specifically colorectal and bladder cancer.

Georgescu, D. et al. [17] conducted a case report for a primarily hepatocellular carcinoma incidentally diagnosed in a patient with nonalcoholic steatohepatitis and post-COVID syndrome. Considering the unfavorable outcomes of this liver condition, the authors highlighted the importance of early diagnosis, recommending regular check-ups in patients with risk factors, such as nonalcoholic fatty liver disease.

In their case report, Lazureanu, D.-C. et al. [18] addressed a rare form of tumor, specifically collision tumors, which occur at the same location but are two different and independent tumor components, expressing unique morphological and immunohistochemical characteristics. This peculiar situation represents a challenge for medical staff, concerning not only the diagnosis but also the realization of therapeutic strategies and the patient’s future outcome.

Belei, O. et al. [19] conducted an infant case report, focusing on the specific features related to neuroendocrine tumors such as vasoactive intestinal peptide (VIP)omas. As a consequence of VIP production, VIPomas result in chronic watery diarrhea, hydroelectrolyte imbalances, and acid–base disorders. This particular case of a child presenting with a retroperitoneal VIPoma posed many diagnostic and therapeutic challenges.

In conclusion, this Special Issue presented many innovative developments in diagnosis and treatment strategies related to digestive disorders, highlighting future important applications with potential for better patient care and improved patient wellbeing.
